# Effect of intensive insulin treatment on plasma levels of lipoprotein-associated phospholipase A_2_ and secretory phospholipase A_2_ in patients with newly diagnosed type 2 diabetes

**DOI:** 10.1186/s12944-016-0368-3

**Published:** 2016-11-23

**Authors:** Xiu-hong Lin, Ming-tong Xu, Jv-ying Tang, Li-fang Mai, Xiao-yi Wang, Meng Ren, Li Yan

**Affiliations:** 1 Department of Clinical Nutrition, Sun Yat-sen Memorial Hospital, Sun Yat-sen University, 107 Yanjiang West Road, Guangzhou, 510120 People’s Republic of China; 2 Department of Endocrinology, Sun Yat-sen Memorial Hospital, Sun Yat-sen University, 107 Yanjiang West Road, Guangzhou, 510120 People’s Republic of China

**Keywords:** Type 2 diabetes, newly diagnosed, Intensive insulin treatment, Atherosclerosis, Lp-PLA_2_, sPLA_2_

## Abstract

**Background:**

China has the highest absolute disease burden of diabetes worldwide. For diabetic patients, diabetes-related vascular complications are major causes of morbidity and mortality. The roles of lipoprotein-associated phospholipase A_2_ (Lp-PLA_2_) and secretory phospholipase A_2_ (sPLA_2_) as inflammatory markers have been recently evaluated in the pathogenesis of both diabetes and atherosclerosis. We aimed to determine the mechanism through which patients with newly diagnosed type 2 diabetes gain long-term vascular benefit from intensive insulin therapy by evaluating the change in Lp-PLA_2_ and sPLA_2_ levels after early intensive insulin treatment and its relevance with insulin resistance and pancreatic β-cell function.

**Methods:**

In total, 90 patients with newly diagnosed type 2 diabetes mellitus were enrolled. All patients received continuous subcutaneous insulin infusion (CSII) for approximately 2 weeks. Intravenous glucose-tolerance test (IVGTT) and oral glucose-tolerance test (OGTT) were performed, and plasma concentrations of Lp-PLA_2_ and sPLA_2_ were measured before and after CSII.

**Results:**

Levels of Lp-PLA_2_ and sPLA_2_ were significantly higher in diabetic patients with macroangiopathy than in those without (*P* < 0.05). After CSII, the sPLA_2_ level decreased significantly in all diabetic patients (*P* < 0.05), while the Lp-PLA2 level changed only in those with macroangiopathy (*P* < 0.05). The area under the curve of insulin in IVGTT and OGTT, the acute insulin response (AIR_3–5_), early phase of insulin secretion (ΔIns30/ΔG30), modified β-cell function index, and homeostatic model assessment for β-cell function (HOMA-β) increased after treatment even when adjusted for the influence of insulin resistance (IR; *P* < 0.001). The HOMA-IR was lower after treatment, and the three other indicators adopted to estimate insulin sensitivity (ISI_ced_, IAI, and QUICKI) were higher after treatment (*P* < 0.05). Correlation analysis showed that the decrease in the Lp-PLA_2_ and sPLA_2_ levels was positively correlated with a reduction in HOMA-IR after CSII (*P* < 0.05). Additionally, multiple linear regression analysis showed that Lp-PLA_2_ and sPLA_2_ independently correlated with HOMA-IR (*P* < 0.05).

**Conclusions:**

Lp-PLA_2_ and sPLA_2_ are closely related to insulin resistance and macroangiopathy in diabetic patients. Intensive insulin therapy might help improve IR and protect against diabetic macroangiopathy by influencing the Lp-PLA_2_ and sPLA_2_ levels.

**Trial registration:**

ChiCTR-TRC-10001618 2010 September 16.

## Background

Currently, China is one of the countries with the highest prevalence of diabetes in Asia and has the largest absolute disease burden of diabetes worldwide. According to the most recent epidemiological survey, the overall prevalence of diabetes was estimated to be 11.6% in Chinese adults [1]. Diabetes-related vascular complications such as cardiovascular disease and stroke are major causes of morbidity and mortality for type 2 diabetic patients (T2DM) [2]. Inflammatory processes have been found to play a role in the pathogenesis of both diabetes and atherosclerosis, and may offer a biological link between the two diseases [3]. Various circulating markers of inflammation have been extensively evaluated for their role as risk predictors of diabetes and atherosclerosis [4–6]. Among these markers, lipoprotein-associated phospholipase A_2_ (Lp-PLA_2_) and secretory phospholipase A_2_ (sPLA_2_) have gained considerable interest in the last decade. Many prospective studies have also indicated that Lp-PLA_2_ and sPLA_2_ are independent predictors of coronary heart disease and carotid stenosis, and they are more closely associated with atherosclerosis as compared to classic inflammatory markers such as C-reactive protein [7, 8].

Lp-PLA_2_ and sPLA_2_ are two subtypes of the PLA_2_ superfamily, a family of enzymes that propagate inflammation by catalyzing the hydrolysis of glycerophospholipids, thereby producing non-esterified fatty acids (FA) such as arachidonic acid. Lp-PLA_2_, also known as platelet-activating factor acetylhydrolase, is a 50-kD Ca^2+^-independent phospholipase and hydrolyzes phospholipids to produce free FAs (FFAs) and lyso-phosphatidycholine, thus promoting inflammation and atherosclerosis. Another important extracellular enzyme of the PLA_2_ superfamily, sPLA_2,_ is a 14-kD Ca^2+^-dependent phospholipase and is normally expressed in arterial walls. Its expression is readily upregulated by inflammatory stimuli, suggesting a potential role in the early phases of response to vessel injury. Most studies have attributed an inflammatory role to Lp-PLA_2_ and sPLA_2_ [9, 10]. In addition, studies suggest that Lp-PLA_2_ is positively associated with insulin resistance and predicts the incidence of T2DM [11]. However, few study has assessed the effect of glycemic status of the patients on sPLA_2_. Moreover, other studies showed that both of Lp-PLA_2_ and sPLA_2_ play critical roles in the development of atherosclerosis and its clinical sequelae [7]. Lp-PLA_2_ and sPLA_2_ are upregulated in atherosclerotic plaques and are strongly expressed in macrophages within the fibrous cap of rupture-prone lesions [7, 12].

Short-term intensive insulin therapy can induce effective glycemic control and improve islet function and insulin sensitivity in patients with newly diagnosed T2DM [13]. In the United Kingdom Prospective Diabetes Study (UKPDS), after a 10-year follow-up, patients with newly diagnosed T2DM who received intensive glucose therapy were found to have a reduced risk of microvascular complications, coronary events, and death from any cause [14]. However, the mechanism through which these patients benefit from intensive insulin therapy is poorly understood. To our knowledge, no study has thus far specifically assessed the effect of intensive insulin therapy on Lp-PLA_2_ and sPLA_2_ levels in patients with newly diagnosed T2DM. Therefore, this study aimed to evaluate the change in Lp-PLA_2_ and sPLA_2_ levels after intensive insulin treatment and the correlation of these two enzymes with insulin resistance and pancreatic β-cell function in patients with newly diagnosed T2DM.

## Methods

### Subjects

Patients with newly diagnosed T2DM were enrolled in this study at the Sun Yat-sen Memorial Hospital of Sun Yat-sen University from October 2010 to March 2012. Diabetes was diagnosed in accordance with the diagnostic criteria of American Diabetes Association (ADA, 2010). All patients were 30–60 years of age, negative for pancreatic islet autoantibody, and had never taken hypoglycemic drugs. Patients with acute hyperglycemic complications, severe hepatic disease, renal dysfunction, cardiac dysfunction, and acute or chronic infections were excluded.

### Research design

This was a self-control study of patients with newly diagnosed T2DM. All the enrolled diabetic patients were admitted to the hospital. The same researcher measured the height, weight, waist circumference, and blood pressure of the subjects on an empty stomach using consolidated tools. Body mass index (BMI) was calculated as weight divided by the square of the height (kg/m^2^). After an overnight fast, venous blood samples were drawn for measuring blood glucose, hemoglobin A1c (HbA1c), lipid profiles (including total cholesterol [TC], triglyceride [TG], high-density lipoprotein cholesterol [HDL-C], and low-density lipoprotein cholesterol [LDL-C]), traditional inflammatory markers (including white blood cells [WBC], FFA, and high-sensitivity C-reactive protein [hsCRP]), Lp-PLA_2_, and sPLA_2_. Subsequently, the patients underwent an intravenous glucose-tolerance test (IVGTT) with 50 mL of 50% glucose solution using the standard protocol. Blood samples were collected before and 3, 5, 7, and 10 min after the injection to assess the glycemic and insulin status. The next day, patients underwent the oral glucose-tolerance test (OGTT) with 75-g glucose load. To determine the glycemic and insulin status, blood samples were obtained before and 30, 60, 120, and 180 min after oral administration.

Thereafter, the patients received insulin lyspro (Humalog Lilly, USA) via an insulin-infusion pump (MiniMed 712) for approximately 2 weeks. The initial insulin doses were 0.4–0.6 IU/kg/day. Total daily doses were divided into 50% basal and 50% bolus injection. The doses of bolus and basal insulin infusions were adjusted according to the capillary blood glucose levels. Patients were asked to monitor their glycemic levels before and 2 h after each meal, and at bedtime. The target blood glucose level was defined as fasting plasma glucose (FPG) level < 6.1 mmol/L and 2-h postprandial plasma glucose (2hPG) level < 7.8 mmol/L. A severe hypoglycemic episode (PG <2.8 mmol/L) was defined as an event requiring the assistance of another person to actively administer carbohydrate, glucagon, or other resuscitative treatments. No oral hypoglycemic agents, antiplatelet medicine, or lipid drugs were administered during the study. Antihypertensive drugs affecting the renin-angiotensin system were also avoided in patients with complications of hypertension. Twenty-four hours after completion of the treatment, all the measurements mentioned above were reviewed.

### Laboratory analyses

For plasma samples, blood was collected in EDTA vials and plasma was separated by centrifugation at 1000 × g for 10 min. The plasma samples were stored at −80°C and analyzed later for the mass of Lp-PLA_2_ and sPLA_2_. Concentrations of Lp-PLA_2_ and sPLA_2_ were assayed using a double-antibody sandwich enzyme-linked immunosorbent assay kit (Uscn Life Science, Peking, China). Three repetitive experiments showed that the average of coefficient of variation within-run was below 5%.

The plasma glucose level was determined by the glucose-oxidase method using a semi-automatic biochemistry analyzer (GF-D200, Shandong, China). The HbA1c concentration was measured using high-pressure liquid chromatography (BIO-RAD, California, USA). The direct chemiluminescence method was used to measure serum insulin levels with an automatic biochemistry analyzer (Immulite 2000, DPC, USA). The lipid profiles were measured by enzymatic colorimetry using a semi-automatic biochemistry analyzer (GF-D200, Shandong, China). Homeostasis model assessment (HOMA) was used to estimate insulin resistance (HOMA-IR) and β-cell function (HOMA-β) as follows: HOMA-IR = FPG × fasting insulin (FINS)/22.5 and HOMA-β = 20 × FINS/(FPG - 3.5). Other indicators used to evaluate β-cell function were as follows. The acute insulin response (AIR) was used to assess the first-phase insulin secretion, including the average increase in plasma insulin levels between the third or fifth minute and the baseline (AIR_3–5_) during IVGTT. The area under the curve of insulin (AUC_Ins_) was calculated as the incremental trapezoidal area during the first 10 min as follows: IVGTT AUC_Ins_ = (3 × FINS + 5 × INS_3_ + 4 × INS_5_ + 5 × INS_7_ + 3 × INS_10_)/2. The early phase of insulin secretion was expressed as ∆INS_30_/∆G_30_ = (INS_30_ - FINS)/(G_30_ - FPG). The second-phase of insulin release was indicated as AUC_Ins_ during OGTT and was calculated as OGTT AUC_Ins_ = (FINS + 2 × INS_30_ + 3 × INS_60_ + 4 × INS_120_ + 2 × INS_180_)/4. Modified β-cell function index (MBCI) was calculated as MBCI = (FPG × FINS)/(G_120_ + G_60_ - 7) and was used for comprehensive assessment of β-cell function. Moreover, three other indicators were adopted to estimate insulin sensitivity: IAI, (calculated as 1/[FINS × FPG]), QUICKI (calculated as 1/[lgFPG + lgFINS]), and ISI_ced_ (calculated as MCR/LgMSI, MCR = M/MG, MG = [G_0min_ + G_30 min_ + G_60 min_ + G_120 min_]/4, MSI = [I_0 min_ + I_30 min_ + I_60 min_ + I_120 min_]/4, M = 75000/120 + [G_0 min_ - G_120 min_] × 1.15 × 180 × 0.19 × body weight/120). In addition, all the indicators for β-cell function were divided by HOMA-IR to exclude interference of insulin resistance [15]. Furthermore, ultrasonography and electrocardiography were performed to screen atherosclerosis in the lower limbs (whether femoral artery or posterior tibial artery had definite plaques), cervical vessels (whether carotid intima-media thickness was more than 1.2 mm), and coronary artery (whether ECG had changes of ST-T which indicated myocardial ischemia).

### Statistical analysis

Statistical analysis was performed using SPSS 13.0 software for windows. Values of variables are expressed as mean ± standard deviation (SD) if their data fit the normal distribution and as median (interquartile range) if their data did not fit the normal distribution. Change in the variables after treatment was assessed using a paired *t*-test or Wilcoxon rank sum test. Pearson or Spearman’s rank correlation analysis was used to assess the association between variables. Multiple linear regression analysis was used with HOMA-IR as a dependent variable. Variables that had skewed distributions were logarithmically transformed (lg10). A two-sided value of *P* < 0.05 was considered statistically significant in all analyses.

## Results

### Baseline characteristics

A total of 90 patients were enrolled in our study. The mean age of the patients was 48.11 ± 9.21 years, and the sex ratio (M/F) was 66:24. The FPG, 2hPG, and HbA1c levels were 11.79 ± 3.57 mmol/L, 24.28 ± 5.90 mmol/L, and 11.70% ± 2.40%, respectively. Waist circumference was 87.49 ± 8.26 cm (normal range in Chinese: <85 cm for men and <80 cm for women), and BMI was 24.03 ± 2.93 kg/m^2^ (normal range in Chinese: 18.5 ~ 23.9 kg/m^2^). Blood pressure was (129 ± 14)/(83 ± 10) mmHg. Lipid profiles were as follows: TC was 5.82 ± 1.21 mmol/L, TG was 1.76(1.26, 3.05)mmol/L, HDL-C was 1.18 ± 0.29 mmol/L, and LDL-C was 3.73 ± 0.98 mmol/L. Traditional inflammatory markers like hsCRP, WBC and FFA were 1.84(1.08,3.67)mg/L, 7.62 ± 2.02 × 10^9^/L, 607.48 ± 225.00 umol/L, respectively. Tests for diabetic angiopathy showed that 14, 9, and 6 patients had atherosclerosis in the lower limbs, cervical vessels, and coronary artery, respectively (including two patients with atherosclerosis both in the lower limbs and cervical vessels and one patient with atherosclerosis both in the lower limbs and coronary artery).

### Changes in the clinical features of diabetic patients after intensive insulin treatment

Ideal glucose control was attained in 4.1 ± 2.0 days and was maintained for 10.0 ± 2.4 days. The maximum dosage of insulin administered was 50.32 ± 13.23 U/day (0.72 [0.64, 0.89] U/kg/day). The frequency of hypoglycemia (≤3.9 mmol/L) during CSII treatment was 2 (1, 3) times. There were no severe hypoglycemic (<2.8 mmol/L) episodes during the short-term intensive interventions. After treatment, the measures of waist circumference, blood pressure, HbA1c, AUC of glucose (AUC_Glu_) of IVGTT and OGTT, TC, TG, LDL-C, and inflammatory markers (including WBC and FFA, but not hsCRP) decreased significantly (*P* < 0.05), whereas the levels of HDL-C and AUC_Ins_ of IVGTT and OGTT increased significantly after CSII (*P* < 0.05) (Table 1).

**Table 1:** Clinical features of 90 newly diagnosed type 2 diabetic patients at baseline and after CSII

	Baseline	After CSII	kind of test	*P*
Waist circumference (cm)	87.49 ± 8.26	86.80 ± 7.93	paired *t*-test	0.021
Body mass index (kg/m^2^)	24.03 ± 2.93	23.85 ± 2.90	paired *t*-test	0.149
Systolic pressure (mmHg)	129 ± 14	121 ± 11	paired *t*-test	<0.01
Diastolic pressure (mmHg)	83 ± 10	76 ± 7	paired *t*-test	<0.01
HbA1c (%)	11.70 ± 2.40	9.82 ± 2.02	paired *t*-test	<0.01
IVGTT AUC_Glu_	89.43 ± 17.38	67.01 ± 11.74	paired *t*-test	<0.01
OGTT AUC_Glu_	79.57 ± 18.88	46.49 ± 9.28	paired *t*-test	<0.01
Total cholesterol (mmol/L)	5.82 ± 1.21	4.89 ± 0.95	paired *t*-test	<0.01
Triglyceride (mmol/L)	1.76 (1.26,3.05)	1.74 (1.29,2.24)	Wilcoxon rank sum test	0.026
HDL-C (mmol/L)	1.18 ± 0.29	1.23 ± 0.29	paired *t*-test	0.035
LDL-C (mmol/L)	3.73 ± 0.98	2.93 ± 0.77	paired *t*-test	<0.01
hsCRP (mg/L)	1.84 (1.08,3.67)	1.69 (0.77,2.96)	Wilcoxon rank sum test	0.54
White blood cells (×10^9^/L)	7.62 ± 2.02	6.75 ± 1.70	paired *t*-test	<0.01
Free fatty acid (umol/L)	607.48 ± 225.00	469.11 ± 171.41	paired *t*-test	0.004
HOMA-IR	1.45 (1.01, 2.45)	0.96 (0.68, 1.85)	Wilcoxon rank sum test	<0.01
IAI	0.03 (0.02, 0.04)	0.05 (0.02, 0.06)	Wilcoxon rank sum test	<0.01
QUICKI	0.67 ± 0.12	0.74 ± 0.15	paired *t*-test	<0.01
ISI_ced_	5.89 (4.33, 7.26)	9.68 (8.25, 11.69)	Wilcoxon rank sum test	<0.01
HOMA-β	6.87 (4.40, 15.73)	21.00 (12.90,43.04)	Wilcoxon rank sum test	<0.01
AIR_3–5_	1.38 (0, 5.25)	3.80 (0.87,7.46)	Wilcoxon rank sum test	<0.01
∆Ins30/∆G30	0.19 (0, 1.01)	1.80 (0.60, 3.29)	Wilcoxon rank sum test	<0.01
MBCI	0.91 (0.73, 1.70)	1.12 (0.74, 2.15)	Wilcoxon rank sum test	0.048
IVGTT AUC_Ins_	11.91 (8.00,29.05)	25.64 (10.32,46.79)	Wilcoxon rank sum test	<0.01
OGTT AUC_Ins_	27.60 (12.20,64.37)	75.45 (46.37,123.53)	Wilcoxon rank sum test	<0.01
HOMA-β/HOMA-IR	4.84 (2.60, 9.59)	20.99 (12.50,31.78)	Wilcoxon rank sum test	<0.01
AIR_3–5_/HOMA-IR	0.95 (0, 2.86)	2.49 (0.45, 6.22)	Wilcoxon rank sum test	<0.01
∆Ins30/(∆G30× HOMA-IR)	0.16 (0, 0.37)	1.11 (0.50, 2.75)	Wilcoxon rank sum test	<0.01
MBCI/HOMA-IR	0.71 (0.55, 0.92)	1.16 (1.01,1.34)	Wilcoxon rank sum test	<0.01
IVGTT AUC_Ins_/ HOMA-IR	9.98 (6.58, 14.82)	21.25 (12.62,31.92)	Wilcoxon rank sum test	<0.01
OGTT AUC_Ins_/HOMA-IR	17.91 (10.01,35.70)	64.35 (47.82,93.13)	Wilcoxon rank sum test	<0.01
Lp-PLA_2_ (ng/ml)	102.9 (76.34,134.31)	88.35 (76.74,125.18)	Wilcoxon rank sum test	0.087
sPLA_2_ (ng/ml)	219.33 (130.03,337.30)	173.78 (80.95,278.09)	Wilcoxon rank sum test	<0.01

*Abbreviations*: *HbA1c* hemoglobin A1c, *IVGTT AUC*_*Glu*_ the areas under the curve of glucose in intravenous glucose tolerance test, *OGTT AUC*_*Glu*_ the areas under the curve of glucose in oral glucose tolerance test, *HDL-C* high density lipoprotein cholesterol, *LDL-C* low density lipoprotein cholesterol, *hsCRP* high sensitivity C reactive protein; *HOMA-IR*, *IAI*, *QUICKI* and *ISI*_*ced*_ are indicators of insulin sensitivity (HOMA-IR = FPG × FIns/22.5; IAI = 1/(FIns × FPG); QUICKI = 1/(lgFPG + lgFIns); ISI_ced_ = MCR/LgMSI, MCR = M/MG, MG = (FPG + Glu30 min + Glu60 min + Glu120 min)/4, MSI = (FIns + Ins30 min + Ins60 min + Ins120 min)/4, M = 75000/120 + (FPG - Glu120 min) × 1.15 × 180 × 0.19 × body weight/120), *HOMA-β*, *AIR*_*3–5*_ ∆Ins30/∆G30, MBCI, IVGTT AUC_Ins_ and OGTT AUC_Ins_ are indicators of island function (HOMA-β = 20 × FIns/(FPG - 3.5); AIR_3–5_ is calculated as the average increase of plasma insulin between the 3rd or 5th minute and the base line in IVGTT; ∆Ins30/∆G30 = (Ins30min - FIns)/(Glu30min - FPG); MBCI = (FPG × FIns)/(Glu120min + Glu60min - 7); AUCIns, the area under the curve (AUC) of insulin, IVGTT AUCIns = (3 × FINS+ 5 × INS3+ 4 × INS5+ 5 × INS7+ 3 × INS10)/2, OGTT AUCIns = (FINS + 2 × INS30+ 3 × INS60+ 4 × INS120+ 2 × INS180)/4; all the indicators regarding to β cell function were respectively divided by HOMA-IR to guarantee from interference of insulin resistance), *Lp-PLA*_*2*_ lipoprotein-associated phospholipase A_2_, *sPLA*_*2*_ secretory phospholipase A_2_

Data are expressed as median (interquartile range) or mean ± SD

Values of *P* < 0.05 are statistically significant

**Fig. 1:**
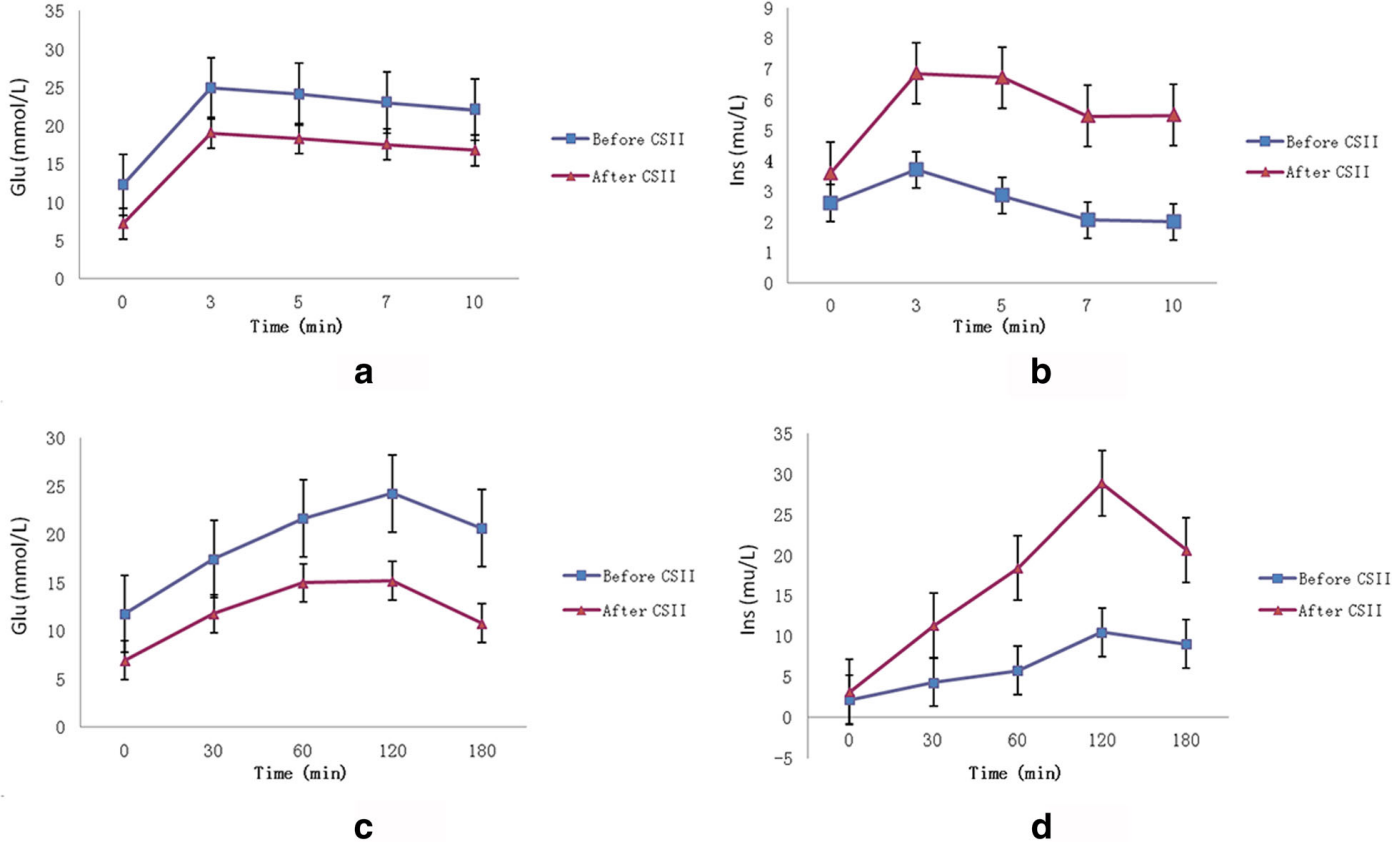
Concentrations of blood glucose and insulin during IVGTT and OGTT before and after CSII (*n* = 90). **a** Means ± SD for glucose concentrations during IVGTT before (■) and after CSII (▲). The AUC_Glu_ in IVGTT decreased significantly (89.43 ± 17.38 mmol · min vs. 67.01 ± 11.74 mmol · min, *P* < 0.01) after CSII. **b** Median (interquartile range) for insulin concentrations during IVGTT before (■) and after CSII (▲). The AUC_Ins_ in IVGTT was significantly elevated (11.91 [8.00, 29.05] mU · min vs. 25.64 [10.32, 46.79] mU · min, *P* < 0.01) after CSII. **c** Means ± SD for glucose concentrations during OGTT before (■) and after CSII (▲). AUC_Glu_ in OGTT decreased significantly (79.57 ± 18.88 mmol · min vs. 46.49 ± 9.28 mmol · min, *P* < 0.01) after CSII. **d** Median (interquartile range) for insulin concentrations during OGTT before (■) and after CSII (▲). AUC_Ins_ in OGTT was significantly elevated (27.60 [12.20, 64.37] mU · min vs. 75.45 [46.37, 123.53] mU · min, *P* < 0.01) after CSII. Values of *P* < 0.05 are statistically significant. CSII, continuous subcutaneous insulin infusion; IVGTT, intravenous glucose-tolerance test; OGTT, oral glucose-tolerance test; SD, standard deviation; AUC_Glu_, area under the curve of glucose; AUC_Ins_, area under the curve of insulin

With regard to the insulin-secretory function of islets, none of the diabetic patients showed first-phase insulin secretion, and the peak of second-phase secretion appeared later than it normally does (120 min vs. 60 min, respectively). After 2 weeks of CSII, the levels of insulin at each time point in both IVGTT and OGTT were significantly elevated (*P* < 0.001) and the first-phase insulin secretion was recovered, whereas the peak of second-phase secretion was delayed (Fig. 1). Indicators of β-cell function including AIR_3–5_, ΔINS_30_/ΔG_30,_ HOMA-β, and MBCI significantly improved after treatment (*P* < 0.05). The results remained the same when they were divided by HOMA-IR to exclude the influence of insulin resistance (*P* < 0.001) (Table 1).

With respect to insulin resistance, the HOMA-IR decreased significantly after treatment, but the IAI, QUICKI, and ISI_ced_ significantly improved after treatment (*P* < 0.001) (Table 1).

### Plasma levels of Lp-PLA_2_ and sPLA_2_ in diabetic patients and their changes after CSII

**Fig. 2:**
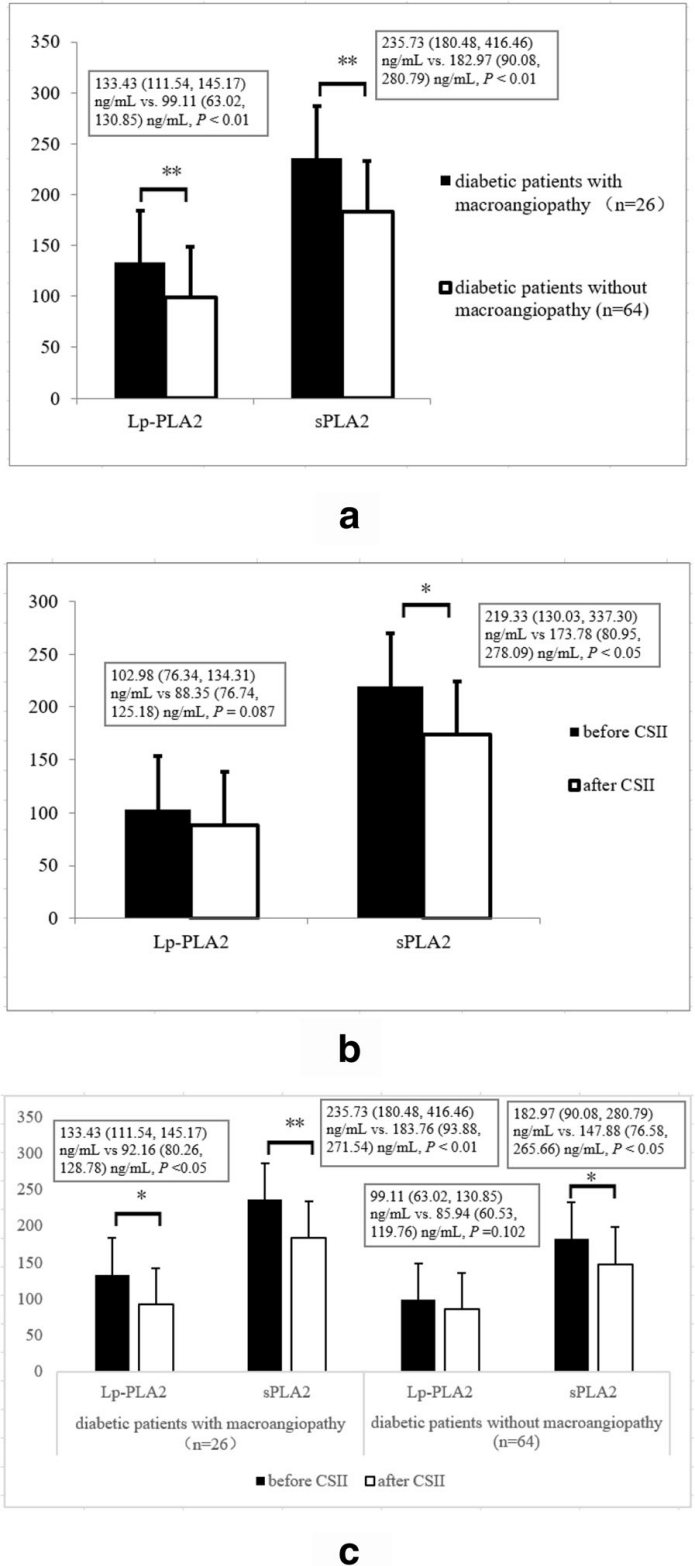
Comparison of the Lp-PLA_2_ and sPLA_2_ levels between diabetic patients with macroangiopathy and those without macroangiopathy (133.43 [111.54, 145.17] ng/mL vs. 99.11 [63.02, 130.85] ng/mL, 235.73 [180.48, 416.46] ng/mL vs. 182.97 [90.08, 280.79] ng/mL, respectively) (**a**); changes of the Lp-PLA_2_ and sPLA_2_ levels after continuous subcutaneous insulin infusion (CSII) in all newly diagnosed type 2 diabetes (102.98 [76.34, 134.31] ng/mL vs. 88.35 [76.74, 125.18] ng/mL, 219.33 [130.03, 337.30] ng/mL vs. 173.78 [80.95, 278.09] ng/mL, respectively, *n* = 90) (**b**); changes of the Lp-PLA_2_ and sPLA_2_ levels after CSII in diabetic patients with or without macroangiopathy (Lp-PLA_2_: 133.43 [111.54, 145.17] ng/mL vs 92.16 [80.26, 128.78] ng/mL, *P* <0.05; 99.11 [63.02, 130.85] ng/mL vs. 85.94 [60.53, 119.76] ng/mL, *P* =0.102; sPLA_2_: 235.73 [180.48, 416.46] ng/mL vs. 183.76 [93.88, 271.54] ng/mL, *P* < 0.01; 182.97 [90.08, 280.79] ng/mL vs. 147.88 [76.58, 265.66] ng/mL, *P* < 0.05) (**c**). *P < 0.05, ** *P* < 0.01. Values of *P* < 0.05 are statistically significant

The plasma levels of Lp-PLA_2_ and sPLA_2_ were 102.98 (76.34, 134.31) ng/mL and 219.33 (130.03, 337.30) ng/mL, respectively, in diabetic patients before CSII. In addition, the plasma levels of Lp-PLA_2_ and sPLA_2_ in diabetic patients with evidence of atherosclerosis were significantly higher than those in diabetic patients without evidence of atherosclerosis (Lp-PLA_2_ levels: 133.43 [111.54, 145.17] ng/mL vs. 99.11 [63.02, 130.85] ng/mL; sPLA_2_ levels: 235.73 [180.48, 416.46] ng/mL vs. 182.97 [90.08, 280.79] ng/mL, respectively, *P* < 0.001) (Fig. 2a). The differences within and among product batches were <10 and <12%, respectively.

The plasma sPLA_2_ level was significantly reduced after CSII in diabetic patients (from 219.33 [130.03, 337.30] ng/mL to 173.78 [80.95, 278.09] ng/mL; *P <* 0.05) (Table 1, Fig. 2b), regardless of whether they had macroangiopathy or not (from 235.73 [180.48, 416.46] ng/mL to 183.76 [93.88, 271.54] ng/mL, *P* < 0.01; and from 182.97 [90.08, 280.79] ng/mL to 147.88 [76.58, 265.66] ng/mL, *P* < 0.05) (Fig. 2c). However, we did not find any statistical difference in the plasma Lp-PLA_2_ level after treatment in all diabetic patients (from 102.98 [76.34, 134.31] ng/mL to 88.35 [76.74, 125.18] ng/mL, *P =* 0.087) (Table 1, Fig. 2b). Further analysis showed that the decrease of Lp-PLA_2_ level after treatment in diabetic patients with macroangiopathy was statistically significant (from 133.43 [111.54, 145.17] ng/mL to 92.16 [80.26, 128.78] ng/mL, *P* <0.05), while it was not the case in those without macroangiopathy (from 99.11 [63.02, 130.85] ng/mL to 85.94 [60.53, 119.76] ng/mL, *P* >0.05) (Fig. 2c).

### Correlation analysis

In patients with newly diagnosed T2DM before CSII, the Lp-PLA_2_ level was positively correlated with the HbA1c concentration (*r* = 0.283, *P* = 0.020), LDL-C level (*r* = 0.269, *P* = 0.029), and HOMA-IR (*r* = 0.309, *P* = 0.037). Correlation analysis for sPLA_2_ and clinical parameters showed a significant correlation of sPLA_2_ with waist circumference (*r* = 0.243, *P* = 0.043) and HOMA-IR (*r* = 0.506, *P* <0.01). No significant correlation was observed between the Lp-PLA_2_ or sPLA_2_ levels and BMI, blood pressure, TC, TG, HDL-C, all indicators used to evaluate β-cell function, and other inflammatory factors (WBC, FFA, and hsCRP) (Table 2).

**Table 2:** Correlations between Lp-PLA2, sPLA2 and clinical parameters in newly diagnosed type 2 diabetes

	Lp-PLA_2_ (ng/ml)	sPLA_2_ (ng/ml)
Body mass index (kg/m^2^)	*r* = 0.121, *P* = 0.311	*r* = 0.123, *P* = 0.311
Waist circumference (cm)	*r* = 0.023, *P* = 0.846	*r* = 0.243, *P* = 0.043*
Systolic pressure (mmHg)	*r* = 0.080, *P* = 0.460	*r* = 0.046, *P* = 0.677
Diastolic pressure (mmHg)	*r* = 0.077, *P* = 0.472	*r* = 0.079, *P* = 0.467
HbA1c (%)	*r* = 0.283, *P* = 0.020*	*r* = 0.076, *P* = 0.545
Total cholesterol (mmol/L)	*r* = 0.224, *P* = 0.070	*r* = 0.147, *P* = 0.247
Triglyceride (mmol/L)	*r* = 0.106, *P* = 0.448	*r* = 0.078, *P* = 0.584
HDL-C (mmol/L)	*r* = 0.240, *P* = 0.072	*r* = 0.176, *P* = 0.199
LDL-C (mmol/L)	*r* = 0.269, *P* = 0.029*	*r* = 0.177, *P* = 0.162
HOMA-IR	*r* = 0.309, *P* = 0.037*	*r* = 0.506, *P* < 0.01**
HOMA-β	*r* = −0.116, *P* = 0.353	*r* = −0.050, *P* = 0.670
IVGTT AUC_Ins_	*r* = −0.012, *P* = 0.294	*r* = −0.038, *P* = 0.742
IVGTT AUC_PG_	*r* = 0.120, *P* = 0.338	*r* = 0.028, *P* = 0.809
AIR_3–5_	*r* = −0.029, *P* = 0.815	*r* = −0.006, *P* = 0.961
△Ins30/△G30	*r* = −0.023, *P* = 0.855	*r* = −0.055, *P* = 0.633
OGTT AUC_Ins_	*r* = −0.088, *P* = 0.483	*r* = −0.055, *P* = 0.635
OGTT AUC_PG_	*r* = 0.049, *P* = 0.688	*r* = 0.042, *P* = 0.709
MBCI	*r* = −0.099, *P* = 0.427	*r* = −0.206, *P* = 0.073
White blood cells (×10^9^/L)	*r* = 0.137, *P* = 0.262	*r* = 0.029, *P* = 0.796
Free fatty acid (umol/L)	*r* = 0.167, *P* = 0.184	*r* = 0.096, *P* = 0.408
hsCRP (mg/L)	*r* = 0.023, *P* = 0.852	*r* = 0.149, *P* = 0.189

*Abbreviations*: *HbA1c* hemoglobin A1c, *IVGTT AUC*_*Glu*_ the areas under the curve of glucose in intravenous glucose tolerance test, *OGTT AUC*_*Glu*_ the areas under the curve of glucose in oral glucose tolerance test, *HDL-C* high density lipoprotein cholesterol, *LDL-C* low density lipoprotein cholesterol, *hsCRP* high sensitivity C reactive protein; *HOMA-IR*, *IAI*, *QUICKI* and *ISI*_*ced*_ are indicators of insulin sensitivity (HOMA-IR = FPG × FIns/22.5; IAI = 1/(FIns × FPG); QUICKI = 1/(lgFPG + lgFIns); ISI_ced_ = MCR/LgMSI, MCR = M/MG, MG = (FPG + Glu30 min + Glu60 min + Glu120 min)/4, MSI = (FIns + Ins30 min + Ins60 min + Ins120 min)/4, M = 75000/120 + (FPG - Glu120 min) × 1.15 × 180 × 0.19 × body weight/120), *HOMA-β*, *AIR*_*3–5*_ ∆Ins30/∆G30, MBCI, IVGTT AUC_Ins_ and OGTT AUC_Ins_ are indicators of island function (HOMA-β = 20 × FIns/(FPG - 3.5); AIR_3–5_ is calculated as the average increase of plasma insulin between the 3rd or 5th minute and the base line in IVGTT; ∆Ins30/∆G30 = (Ins30min - FIns)/(Glu30min - FPG); MBCI = (FPG × FIns)/(Glu120min + Glu60min - 7); AUCIns, the area under the curve (AUC) of insulin, IVGTT AUCIns = (3 × FINS+ 5 × INS3+ 4 × INS5+ 5 × INS7+ 3 × INS10)/2, OGTT AUCIns = (FINS + 2 × INS30+ 3 × INS60+ 4 × INS120+ 2 × INS180)/4; all the indicators regarding to β cell function were respectively divided by HOMA-IR to guarantee from interference of insulin resistance), *Lp-PLA*_*2*_ lipoprotein-associated phospholipase A_2_, *sPLA*_*2*_ secretory phospholipase A_2_

Values of *P* < 0.05 are statistically significant

**Fig. 3:**
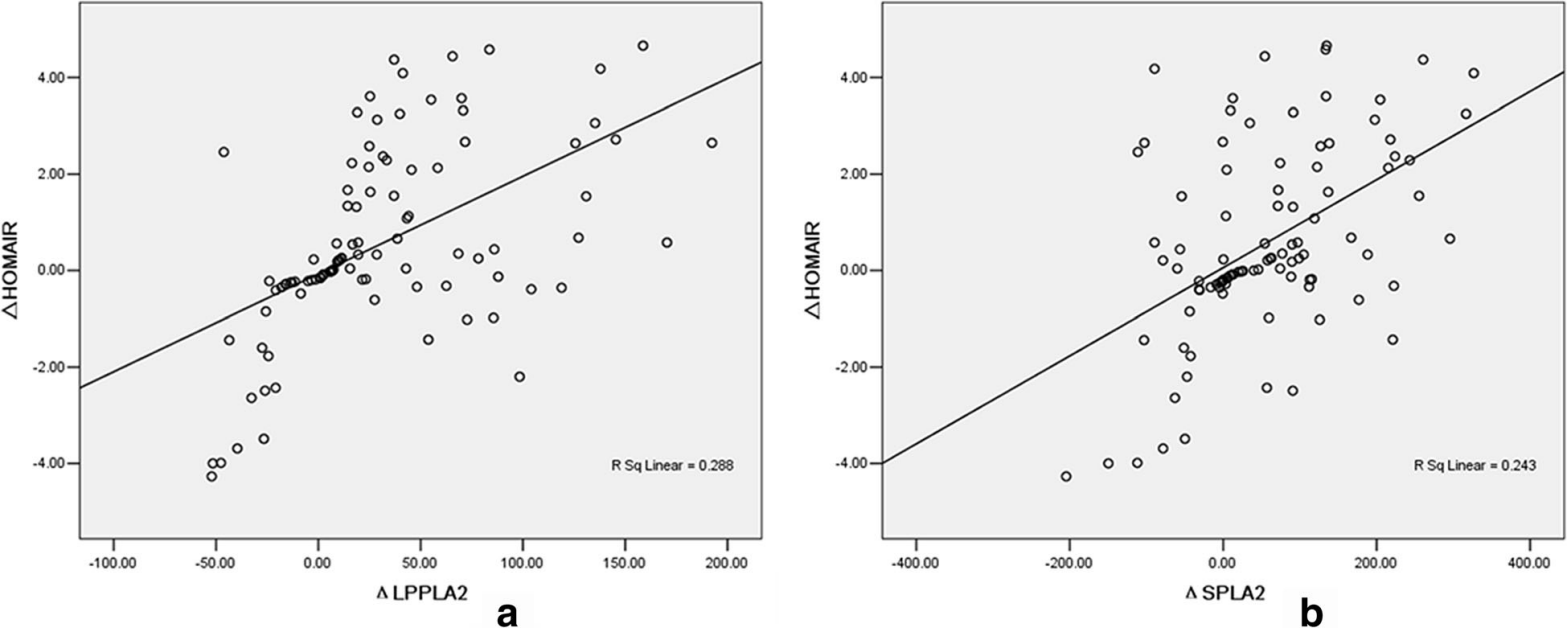
Correlation between ΔLp-PLA_2_ and ΔHOMA-IR in diabetic patients after CSII (*r* = 0.590, *P* < 0.05) (**a**); correlation between ΔsPLA_2_ and ΔHOMA-IR in diabetic patients after CSII (*r* = 0.476, *P* < 0.05) (**b**). Values of *P* < 0.05 are statistically significant. ΔLp-PLA_2_, change in Lp-PLA_2_ levels; ΔsPLA_2_, change in sPLA_2_ levels; ΔHOMA-IR, change in homeostatic model assessment for insulin resistance

After CSII, the decrease in the Lp-PLA_2_ (ΔLp-PLA2) and sPLA_2_ (ΔsPLA2) levels were both positively correlated with the reduction in HOMA-IR (ΔHOMA-IR) (*r* = 0.590, *P* < 0.05 and *r* = 0.476, *P* < 0.05, respectively) (Fig. 3a, 3b). Further, multiple linear regression analysis showed that both Lp-PLA_2_ and sPLA_2_ levels were independent factors that were correlated with HOMA-IR (β = 0.372 and β = 0.560 respectively, *P* < 0.05).

## Discussion

Early intensive insulin therapy could effectively reduce glucotoxicity and lipotoxicity, protect islet function, and alleviate insulin resistance in patients with newly diagnosed T2DM [13]. Using comprehensive indicators, our study showed that early intensive insulin treatment could not only reduce waist circumference, blood pressure, blood glucose (FPG, 2-h postprandial PG, HbA1c concentration, AUC_Glu_ during both IVGTT and OGTT), blood lipid levels (TG, TC, and LDL-C), levels of traditional inflammatory factors (WBC and FFA), and insulin resistance (HOMA-IR), but also distinctly improve the HDL-C levels, insulin sensitivity (IAI, QUICKI, and ISI_ced_) and β-cell function (HOMA-β, MBCI, AIR_3–5_, ∆Ins_30_/∆G_30_, and AUC_Ins_ during IVGTT and OGTT) in patients with newly diagnosed T2DM.

In the present study, the plasma concentration of Lp-PLA_2_ was positively correlated with the HbA1c concentration in patients with T2DM during the early stages of disease, which may indicate an important role of Lp-PLA_2_ in predicting T2DM [11]. The relationship between sPLA_2_ and the risk of diabetes is still unclear, and studies with a larger sample size are needed to confirm this relationship. In addition, Lp-PLA_2_ and sPLA_2_ levels were significantly increased in diabetic patients with macroangiopathy as compared to those without macroangiopathy. This finding is consistent with the view that Lp-PLA_2_ and sPLA_2_ are important risk predictors for atherosclerosis [7, 16]. However, it is uncertain whether plasma Lp-PLA_2_ and sPLA_2_ could be markers of subclinical atherosclerosis for diabetic patients [17, 18]. In our study, we found positive correlations between these two enzymes and several atherogenic factors such as LDL-C, waist circumference, and IR in patients with newly diagnosed diabetes. Therefore, we propose that Lp-PLA_2_ and sPLA_2_ may affect the incidence of atherosclerosis in diabetic patients. A recent study suggested that the relative distribution of Lp-PLA_2_ between LDL and HDL determines its pro- or anti-inflammatory action. According to this assumption, Lp-PLA_2_ is anti-inflammatory when bound to HDL, but pro-inflammatory when bound to LDL [19]. Moreover, an increase in the binding between Lp-PLA_2_ and LDL was observed in diabetic patients [20], which could support the pro-atherosclerotic role of Lp-PLA_2_ in diabetes. However, studies on sPLA_2_ in diabetic patients are lacking in the literature.

Currently, animal experiments and clinical trials involving an Lp-PLA_2_ inhibitor (darapladib) are underway and have been shown to reduce the progression of atherosclerosis in animals [21, 22] but not in humans [23] with or without diabetes. On the other hand, studies on a sPLA_2_ inhibitor (varespladib) showed that administration of 500 mg daily for 8 weeks could significantly reduce the post-ACS (acute coronary syndrome) inflammatory response, especially in diabetic patients [24]. In our study, we aimed to determine whether intensive insulin treatment could reduce the levels of Lp-PLA_2_ and sPLA_2_ in patients with newly diagnosed T2DM. This issue has not been studied previously and may help in resolving the atherosclerotic complications resulting from intensive insulin therapy in these patients. To our knowledge, this study is the first to provide evidence that the plasma concentration of sPLA_2_ is significantly decreased after short-term intensive insulin therapy in patients with newly diagnosed T2DM. The more sensitive change in sPLA_2_ levels could be attributed to its role as an acute-phase protein [25]. Although result of changes in Lp-PLA_2_ after treatment in all diabetic patients was negative—which may be due to the small sample size and short time of therapy or observation—the level of Lp-PLA_2_ was significantly decreased in diabetic patients with macroangiopathy. Considering the close relationship between Lp-PLA_2_ or sPLA_2_ and atherosclerosis, we believe that intensive insulin treatment could alleviate inflammation in diabetic patients by decreasing the levels of Lp-PLA_2_ and sPLA_2_, and such treatment may further prevent or postpone the occurrence and progression of diabetic atherosclerotic complications.

As novel inflammatory markers, both Lp-PLA_2_ and sPLA_2_ seem to be related with IR. Studies have suggested that that the concentrations of Lp-PLA_2_ and sPLA_2_ are high in patients with metabolic diseases accompanied by IR, such as obesity [26, 27] and polycystic ovary syndrome [28]. Kudolo et al. [29] detected the circulating levels of plasma Lp-PLA_2_ in nine patients with obesity, six patients with diabetes, and nine healthy controls and for the first time, they showed that the Lp-PLA_2_ level was significantly correlated with IR. Several studies thereafter observed a similar relation between Lp-PLA_2_ or sPLA_2_ and IR, mainly in people with normal blood glucose [11, 30]. Our study indicated the same observations in patients with newly diagnosed T2DM, and the correlation between sPLA_2_ and IR in diabetic patients was illustrated for the first time. This finding is supported by further results showing that the decrease in both Lp-PLA_2_ and sPLA_2_ levels was positively correlated with the reduction in HOMA-IR after intensive insulin treatment. However, the definite relation of the cause and effect between Lp-PLA_2_ or sPLA_2_ and IR is still unclear. Numerous studies have shown that low-grade chronic inflammation may induce or aggravate IR [31, 32]. Noto et al. [33] found that hydrolysis of oxidized phospholipids by Lp-PLA_2_ produces FFA and lysolecithin, both of which stimulate endothelial cells to generate inflammatory cytokines and ultimately induce IR in both muscle and adipose tissue. Furthermore, Iwase [34] and Huang et al. [35] showed that metformin has the potential to improve IR in a rat model of high-fat diet–induced non-alcoholic fatty liver disease. This effect was due to the decrease in sPLA_2_ levels and inflammation. All the above mentioned studies concluded that Lp-PLA_2_ or sPLA_2_ was responsible for the IR. However, several studies have shown contrasting findings. Kudolo et al. [29] believed that the levels of Lp-PLA_2_ in diabetic patients increased as a result of hyperinsulinemia. In addition, Ramanadham et al. [36] indicated that sPLA_2_ is present within insulin secretory granules, and stimulation of the Langerhan’s island with insulin secretagogues leads to the co-secretion of insulin and sPLA_2_. These researchers believe that hyperinsulinemia due to IR increases the Lp-PLA_2_ or sPLA_2_ levels. Therefore, the causal relationship between Lp-PLA_2_ or sPLA_2_ and IR is still controversial, and further studies are needed to reach a consensus on this issue.

In the current study, we did not observe any correlation between the plasma Lp-PLA_2_ or sPLA_2_ levels and β-cell function, irrespective of whether the influence of IR was adjusted for. Mayer et al. found a positive association between Lp-PLA_2_ activity (not mass) and pancreatic β-cell function [37]. However, studies with a larger sample size are required to establish the association between the plasma Lp-PLA_2_ or sPLA_2_ levels and pancreatic β-cell function.

Despite our important findings, there were several limitations to this study. First, the number of patients enrolled in this study was insufficient and the short period of intervention may have resulted in the absence of a significant change in the Lp-PLA_2_ level after treatment. Second, the Lp-PLA_2_ and sPLA_2_ levels in patients with microvascular complications were not discussed because only a few studies have thus far focused on the relationship between microangiopathy and Lp-PLA_2_ or sPLA_2_.

## Conclusions

In summary, the plasma Lp-PLA_2_ and sPLA_2_ levels are increased in patients with newly diagnosed T2DM and macroangiopathy as compared to those with newly diagnosed T2DM but without macroangiopathy. Early intensive insulin therapy can be effectively used to achieve adequate glycemic control, improve β-cell function and IR, and decrease plasma Lp-PLA_2_ and sPLA_2_ levels in patients with newly diagnosed T2DM, especially in those with macroangiopathy. The change in both the Lp-PLA_2_ and sPLA_2_ levels after CSII were positively correlated with the change in HOMA-IR, and Lp-PLA_2_ and sPLA_2_ levels are both independent factors that are positive correlated with IR in patients with T2DM. Therefore, the decrease in Lp-PLA_2_ and sPLA_2_ levels by intensive insulin therapy might contribute to the improvement of IR as well as protect from diabetic atherosclerotic complications.

## Abbreviations

2hPG: Postprandial 2-h plasma glucose
ADA: American Diabetes Association
AIR: Acute insulin response
AUC: Area under the curve
AUC_Glu_: Area under the curve of glucose
AUC_Ins_: Area under the curve of insulin
BMI: Body mass index
CSII: Continuous subcutaneous insulin infusion
FFA: Free fatty acid
FPG: Fasting plasma glucose
HbA1c: Hemoglobin A1c
HDL-C: High density lipoprotein cholesterol
hsCRP: High sensitivity C-reactive protein
IR: Insulin resistance
IVGTT: Intravenous glucose-tolerance test
LDL-C: Low density lipoprotein cholesterol
Lp-PLA_2_: Lipoprotein-associated phospholipase A_2_
OGTT: Oral glucose-tolerance test
sPLA_2_: Secretory phospholipase A_2_
TC: Total cholesterol
TG: Triglyceride
WBC: White blood cells
